# Maintenance of pluripotency in mouse ES cells without *Trp53*

**DOI:** 10.1038/srep02944

**Published:** 2013-10-15

**Authors:** Masaki Shigeta, Satoshi Ohtsuka, Satomi Nishikawa-Torikai, Mariko Yamane, Setsuko Fujii, Kazuhiro Murakami, Hitoshi Niwa

**Affiliations:** 1 Laboratory for Pluripotent Stem Cell Studies, RIKEN Center for Developmental Biology (CDB), 2-2-3 Minatojima-minamimachi, Chuo-ku, Kobe 6500047, Japan; 2 Laboratory for Development and Regenerative Medicine, Kobe University Graduate School of Medicine, 7-5-1 Kusunokicho, Chuo-ku, Kobe, Hyogo 6500017, Japan; 3 CREST, Sanbancho, Chiyoda-ku, Tokyo, 1020075, Japan

## Abstract

Tumor suppressor Trp53 works as a guardian of the genome in somatic cells. In mouse embryonic stem (ES) cells, it was reported that Trp53 represses pluripotency-associated transcription factor *Nanog* to induce differentiation. However, since *Trp53*-null mice develop to term, *Trp53* is dispensable for both the maintenance and differentiation of the pluripotent stem cell population *in vivo*, suggesting the differential functions of Trp53 in ES cells and embryos. To reveal the basis of this discrepancy, here we established a new line of *Trp53*-null ES cells by sequential gene targeting and evaluated their ability to differentiate *in vitro* and *in vivo*. We found that *Trp53*-null ES cells had defects in differentiation *in vitro* as reported previously, whereas they were able to contribute to normal development in chimeric embryos. These data indicated that the requirement of *Trp53* for maintaining and executing the ES pluripotency is not absolute.

Maintenance of the genome integrity in cells is important for keeping homeostasis of multi-cellular organisms. Tumor suppressor Trp53 is one of the most important components to protect the genome from the oncogenic mutations. It controls cell-cycle arrest, apoptosis and stem cell differentiation by activating and repressing its downstream targets^1^,^2^. Trp53 mainly acts as a transcription factor to activate and repress the target gene expressions. It is expressed ubiquitously in somatic cells and normally its protein product Trp53 is in rapid turnover by active degradation mediated by the E3 ubiquitin ligase Mdm2 or Mdmx. Induction of the DNA damage induces inactivation of Mdm2 that results in accumulation of Trp53 and its nuclear localization. Nuclear localized Trp53 causes arrest of cell-cycle progression and apoptosis to eliminate the cells with damaged genome from the organisms^3^.

Mouse embryonic stem (ES) cells are pluripotent stem cells derived from the inner cell mass of the blastocyst-stagte embryos^4^,^5^. They continue self-renewal in the optimal culture condition *in vitro*, which commonly contain the cytokine leukemia inhibitory factor (LIF) as a repressor of differentiation^6^. Even after a prolonged culture, their pluripotency is maintained as confirmed by injection of these ES cells into blastocyst that give rise to chimeric embryos in which ES cell-derived cells contribute to all germ layers including germ cells^7^. It was reported that Trp53 functions in a unique mode in mouse ES cells^2^. Trp53 is expressed in mouse ES cells, localized in cytoplasm and degradated in a Mdm2/Mdmx-dependent manner as found in other somatic cell types^8^,^9^. Induction of differentiation activates Trp53, which represses the pluripotency-associated transcription factor *Nanog*, suggesting its function to drive differentiation program properly^10^. This process could be regulated by a Trp53 deacetylase Sirt1 by controlling Trp53 subcellular localization^11^ as well as by the expression of a specific isoform of Trp53, delta40p53, in ES cells^12^. Recently, Aurora kinase A was identified as a repressor of Trp53 by phospholyrating it directly, which could also be one of the mechanisms to maintain self-renewal by repressing the differentiation program induced by Trp53^13^.

In contrast to these suggested functions of Trp53 in mouse ES cells, it was known that although Trp53 is activated by DNA damage in mouse ES cells, it has no ability to activate Trp53-mediated DNA damage response such as cell-cycle arrest, apoptosis or senescense as found in somatic cells^14^. This might be due to the unique cell-cycle regulation in mouse ES cells lacking the check point in transition from G1 to S phase^15^. Moreover, *Trp53*-null mice develop normally although they showed high incidence of tumor formation, indicating that the function of *Trp53* is dispensable for self-renewal and differentiation of pluripotent stem cells transiently appeared in the developmental process^16^. Why does the requirement of *Trp53* in differentiation of pluripotent stem cells look different between embryos and ES cells? The distinct role of the LIF signaling in ES cells and embryo has been well analyzed: ES cells require the activation of Stat3 by LIF for continuous self-renewal in serum-containing culture condition^17^ while *Stat3*-null embryos keep pluripotent cell population in pre- and early post-implantation development^18^. Interestingly, *Stat3*-null ES cells are able to be established in a defined serum-free culture condition without LIF^19^, indicating the context-dependent requirement of *Stat3* function in ES cells. How about in the case of *Trp53*? It may be absolutely required in ES cells to execute the differentiation program. Alternatively, the requirement of *Trp53* may be context-dependent and thus dispensable for differentiation of ES cells in the context of embryonic development, i.e. the context in which chimeric embryos from *Trp53*-null ES cells are developed as *Trp53*-null embryos. Since the ability of *Trp53*-null ES cells to contribute chimeric embryos has never been assessed, here we established a new line of *Trp53*-null ES cells and tested their character *in vitro* and *in vivo*. We revealed that *Trp53*-null ES cells retain the ability to contribute to chimeric embryos although they showed abnormality in differentiation *in vitro*.

## Results

### Transient nuclear localization of Trp53 during differentiation in mouse ES cells

**Figure 1:**
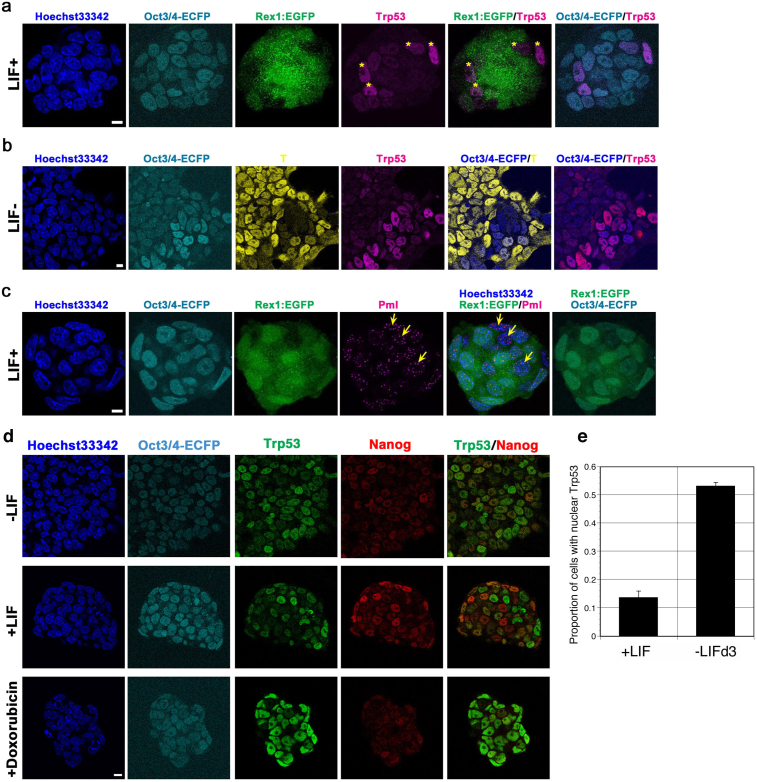
Trp53 expression in undifferentiated and differentiating ES cells. (a) Trp53 expression in undifferentiated ES cells. OCRG9 ES cells expressing Rex1-Egfp and Oct3/4-Ecfp cultured with LIF for 3 days were fixed and immunostained with anti-Trp53 (Alexa 594) and with Hoechst33342 for nuclear staining. Nuclear staining of Trp53 was observed in Oct3/4-Ecfp positive/Rex1-negative or low population (yellow asterisk). Scale bar = 14.5 μm. (b) Trp53 expression in differentiating ES cells. Differentiating ES cells cultured without LIF for 3 days were fixed and immunostained with anti-Trp53 (Alexa 594) and anti-T antibodies (Alexa 555). Trp53 constantly co-localized with Oct3/4-Ecfp, but not with T. Scale bar = 14.5 μm. (c) Pml expression in undifferentiated ES cells. OCRG9 ES cells cultured with LIF for 3 days were fixed and immunostained by anti-Pml antibody (Alexa 594). Larger PML bodies were detected abundantly in some Rex1-negative cells (yellow arrow). Scale bar = 14.5 μm. (d) Expression of Nanog and Trp53 in ES cells. OLC2-1 ES cells carrying Oct3/4-Ecfp were cultured without LIF for 2 days (-LIF: top line), with LIF for 2 days (+LIF; middle line) or with LIF for 2 days followed by treatment with doxorubicin (0.5 μM) for 5 hours and immunostained by anti-Trp53 (Alexa 488) and anti-Nanog (Alexa 594). (e) Proportion of cell carrying nuclear Trp53. The numbers of the cells possessing the strong nuclear Trp53 signal by immunostaining of OCRG9 ES cells cultured with or without LIF for 3 days were counted in three independent wells and the porportion to the total cell numbers were indicated with SD.

It was reported that Trp53 is localized in the cytoplasm and translocates into the nuclei by the induction of DNA damage^8^. However, when we stained mouse ES cells with anti-Trp53 mouse monoclonal antibody 1C12, we found a few cells possessing strong signal of nuclear-localized Trp53 in conventional culture condition, which were 14% of total cells, whereas others had weak signal of nuclear Trp53 (Fig. 1a, 1e). We obtained similar observation with other anti-Trp53 antibody (data not shown). These strong nuclear localized Trp53 population merged with the Oct3/4-positive/Rex1-negative population in OCRG9 ES cell line that carries *Oct3/4-Ecfp* fusion gene and *Rex1*-promoter-driven *Egfp* gene^20^. Since the Oct3/4-positive/Rex1-negative population represents the pluripotent stem cells in the late developmental stage that are ready for undergoing differentiation^21^, these data suggested that the nuclear localization of Trp53 was induced at the initiation of the differentiation event.

To confirm the regulation of Trp53 localization in differentiation process, we tested the localization of Trp53 in ES cells undergoing differentiation by withdrawal of LIF from the culture medium. The mesoderm marker T (also known as *Brachyury*)^22^ was transcriptionally up-regulated by day 4 after the withdrawal of LIF (data not shown) and its nuclear staining was detectable from day 3 heterogeneously by immunostaining (Fig. 1b). In parallel, the pluripotency-associated transcription factor *Oct3/4* was transcriptionally down-regulated after day 2 (data not shown). Trp53 started to accumulate in the nuclei on day 2 (Fig. 1d) and its nuclear localization reached to the maximal level on day 3 (Fig. 1b), which was 53% of total cells (Fig. 1e), although no obvious change was observed in its transcription level during this period (data not shown). Interestingly, Oct3/4 signal, which was retained only in few cells on day 3 after withdrawal of LIF, never merged with T during the differentiation, and Trp53 signal always merged with Oct3/4 but not with T (Fig. 1b), suggesting that the nuclear Trp53 might mark the pluripotent stem cells that are ready to exit the pluripotency and enter into the differentiated state.

To evaluate the transcriptional activity of nuclear localized Trp53 during differentiation, we tested the expression of Pml in self-renewing mouse ES cells since it was reported that *Pml* is a direct target of Trp53^23^. Pml is a component of the macromolecular nuclear structure, PML body. As shown in Fig. 1c, large PML bodies were detected in the Oct3/4-positive/Rex1-negative population as found in the case of the nuclear Trp53 (Fig. 1a), suggesting that the nuclear Trp53 is active in these cells to direct the expression of the target genes. These data indicated that Trp53 is transiently localized in the nuclei in the pluripotent stem cell population during differentiation and is functionally regulating the expression of the target genes.

How about the relationship between Trp53 and Nanog? We tested nuclear localization of Trp53 and Nanog in ES cells carrying the *Oct3/4-Ecfp* fusion gene by co-immunostaining. As shown in Fig. 1d, Nanog was exclusively expressed in Trp53-negative cells in undifferentiated ES cells (+LIF; middle line), which was consistent with our previous observation that Nanog is predominantly expresseed in Rex1-positive population^20^ and with the reciprocal expression of Rex1 and Trp53 shown above. When differentiation was induced by withdrawal of LIF, the expression of Nanog most likely disappeared after day 2 (−LIF; top line) and also the transcriptional level was down-regulated (data not shown) coinciding with the predominant accumulation of Trp53 in the nuclei. When DNA damage was induced in undifferentiated ES cells by the treatment with Doxorubicin for 3 hours, strong nuclear accumulation of Trp53 was observed with loss of Nanog staining although Oct3/4-Ecfp fusion protein remained in the nuclei (+Doxorubicin; bottom line). These data suggest the negative regulation of Nanog by nuclear localized Trp53 as reported previously^10^.

### Aneuploidy in *Trp53*-null ES cells

**Figure 2:**
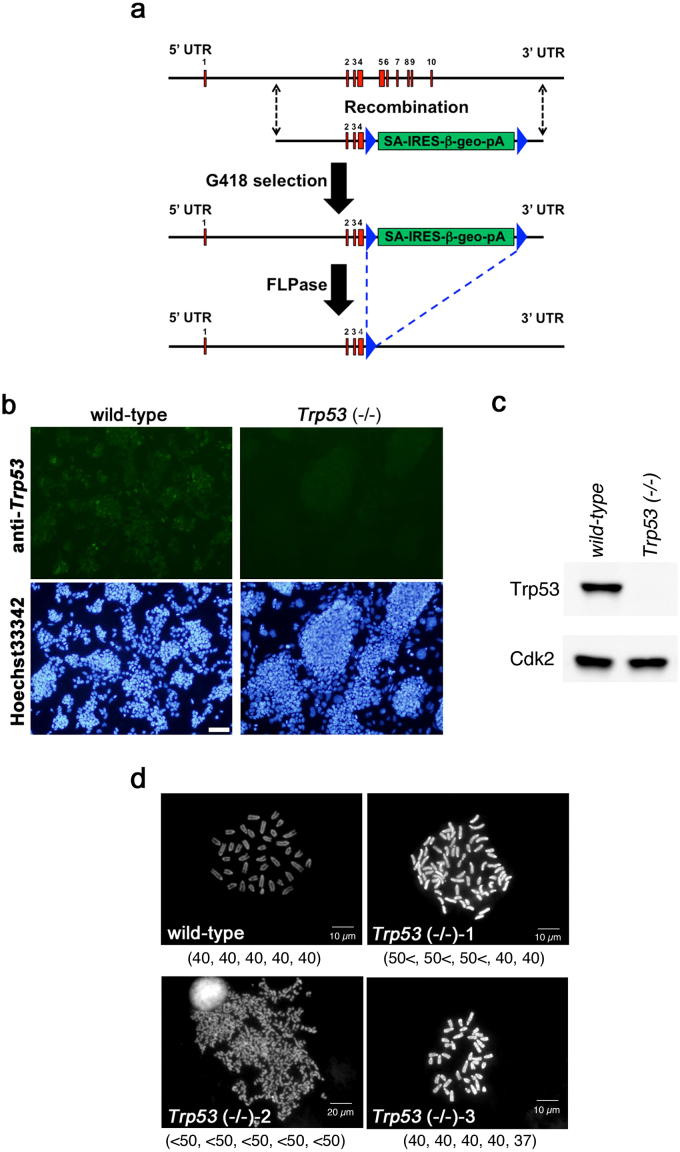
Generation of *Trp53*-null ES cells with normal karyotype. (a) Schematic representation of *Trp53* gene-targeting strategy. Red boxes indicate the exons of *Trp53*. Blue triangles illustrate *Frt* sites flanking *SA-IRES-β-geo-pA* (green box). (b) Immunostaining of wild-type and *Trp53*-null ES cells for Trp53. ES cells were fixed and immunostained with anti Trp53 antibody (Alexa 488). Scale bar = 100 μm. (c) Western blotting of wild-type and *Trp53*-null ES cells for Trp53. Cdk2 acts as a control. (d) Karyotype analyses of *Trp53*-null ES cells. The karyotypes of independently isolated *Trp53*-null ES cells and wild-type ES cells were evaluated by counting metaphase chromosome spreads after Carnoy's fixation as described in Material and Methods. Note that *Trp53*-null ES cell: clone 3 showed normal karyotype. Numbers of chromosomes of 5 samples for each cell lines were indicated below the photomicrographs.

To confirm the function of Trp53 in the differentiation of mouse ES cells, we established a new *Trp53*-null ES cell line by serial gene targeting. Using a promoter-less gene targeting vector to replace the exons encoding the DNA binding domain by *β-geo* (an artificial fusion gene of β-galactosidase and neomycin phosphotransferase)^24^ (Fig. 2a), we obtained heterozygous ES cell lines. After the removal of the *β-geo* cassette flanked by *Frt* sequences using a transient expression of the *FLP* recombinase, we introduced the same knock-out vector into the heterozygous ES cells and selected the homozygous *Trp53*-null ES cells. The proper loss of *Trp53* was confirmed by genotyping using PCR, immunostaining and western blotting with Trp53-antibody, and RT-qPCR (Fig. 2b, 2c, Supplemental Fig. 2 and data not shown).

Since it was known that Trp53 is important as a guardian of the genome stability, we first assessed the karyotypes of the *Trp53*-null ES cells independently isolated from the heterozygous ES cells in which the second *Trp53* allele was correctly disrupted. As a result, we found that many of the *Trp53*-null ES cell lines possessed aneuploidy, but a few of them retained the normal karyotype (Fig. 2d). Interestingly, once the *Trp53*-null ES cell lines with normal karyotype were established, they never showed the genomic instability as reported previously. Therefore, the loss of *Trp53* has a functional impact for keeping normal karyotype but it is able to be compensated, possibly by the function of the other *Trp53*-related genes such as *Trp73*.

### *Trp53*-null ES cells show defects in the induction of differentiation markers after withdrawal of LIF

**Figure 3:**
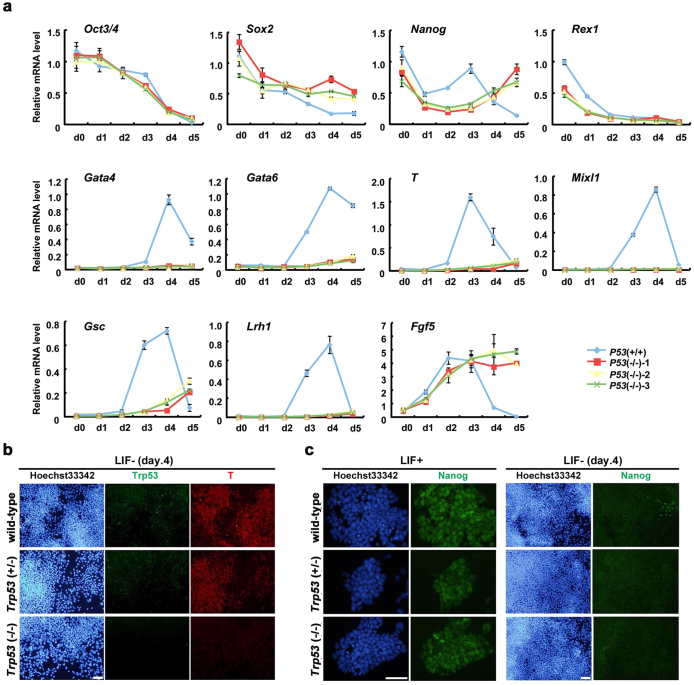
Differentiation of *Trp53*-null ES cells in two-dimensional culture. (a) q-PCR analyses of gene expression profiles during differentiation in wild-type (blue) and three *Trp53*-null (red) ES cells cultured on gelatin-coated dishes without LIF. Transcript levels of indicated genes were evaluated at everyday after withdrawal of LIF for 5 days and normalized to the amount of *Gapdh* mRNA. Results are plotted as the expression level relative to the wild-type at day 0 (set as 1.0) with the mean ± SD of one biological sample assayed in three experiments. (b) Immunostaining of *Trp53*-null ES cells for T. Wild-type, *Trp53* (+/−) and *Trp53* (−/−) ES cells at day 4 after withdrawal of LIF were fixed and stained by anti-Trp53 (Alexa 488) and anti-T antibody (Alexa 594) with Hoechst33342. Scale bar = 100 μm. (c) Immunostaining of *Trp53*-null ES cells for Nanog. ES cells with indicated genotypes cultured with (left panel) or without (right panel) LIF for 4 days were fixed and immunostained by anti-Nanog antibody (Alexa 488) with Hoechst33342. Scale bar = 100 μm.

Previous reports indicated that *Trp53*-null ES cells show defects in up-regulation of the differentiation marker genes and down-regulation of the pluripotency-associated genes^10^. We tested the ability of differentiation in the *Trp53*-null ES cells with normal karyotype that we established. When cultured without LIF, they underwent a morphological differentiation as same as the wild-type and the *Trp53*-heterozygous ES cells (see phase contrast images in Supplemental Fig. 1a) with the change of the cell-cycle profile from ES-specific type to the somatic cell type^25^ monitored by Fucci system^26^ (Supplemental Fig. 1a, 1b). However, when the relative mRNA expression level of the differentiation marker genes were examined by RT-qPCR, we found that the up-regulation of the mesoderm markers *T*, *Mixl1*^27^, *Gsc*^28^ and *Lhx1*^29^, and the extra-embryonic endoderm markers *Gata4* and *Gata6*^30^ were impaired in the *Trp53*-null ES cells (Fig. 3a). This attenuation was also consistent with the negative immunostaining of T and Gata4 at day 4 (Fig. 3b and data not shown). Impaired up-regulation of *T* is consistent with a previous report^10^ although we observed morphological differentiation.

In contrast to the drastic change in the induction of the differentiation markers, the kinetics of down-regulation of the pluripotency-associated genes looked normal (Fig. 3a). Although a previous report mentioned the persistence of *Nanog* expression after induction of differentiation^10^, we observed that *Nanog* was transcriptionally down-regulated properly from day 2 (Fig. 3a), which was also confirmed by immunostaining (Fig. 3c). These data indicated that *Trp53*-null ES cells have deficiencies in the induction of differentiation markers during the differentiation process triggered by withdrawal of LIF but the repression of *Nanog* occurs normaly in the absence of *Trp53*.

### *Trp53*-null ES cells contribute to chimeric embryos

**Figure 4:**
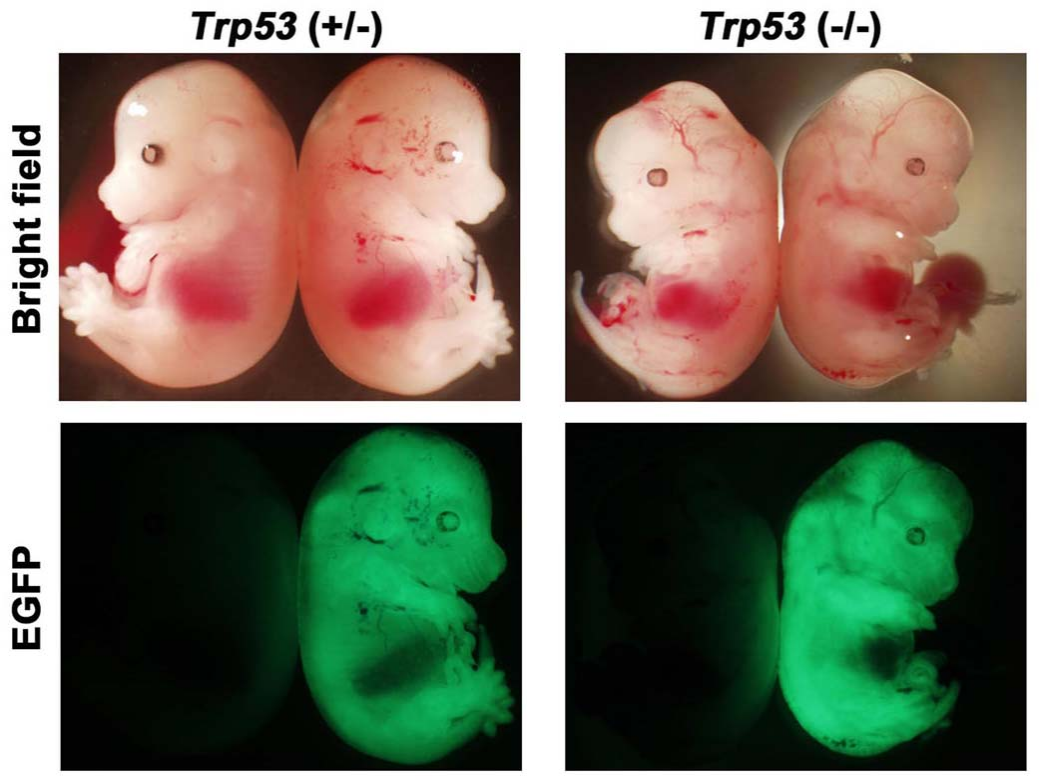
Chimeric embryos with *Trp53*-null ES cells. Chimeric embryos at 14.5 dpc obtained by injection of *Trp53* (+/−) (left) and *Trp53* (−/−) (right) ES cells carrying constitutively-active *Egfp* transgene.

We confirmed that the new *Trp53*-null ES cell lines with normal karyotype show defects in differentiation as reported previously. To test their ability to adopt the normal developmental process, we injected them into the blastocysts to form the chimeric embryos. By the injection of a single *Trp53*-null ES cell into the blastocyst cavity, we succeeded to obtain chimeric embryos in which the ES-derived cells contributed to the three germ layers as efficiently as wild-type ES cells (Fig. 4 and Supplemental Fig. 4). These data clearly demonstrated that *Trp53*-null ES cells retain pluripotency.

### ES cells differentiate normally in embryoid bodies without *Trp53*

**Figure 5:**
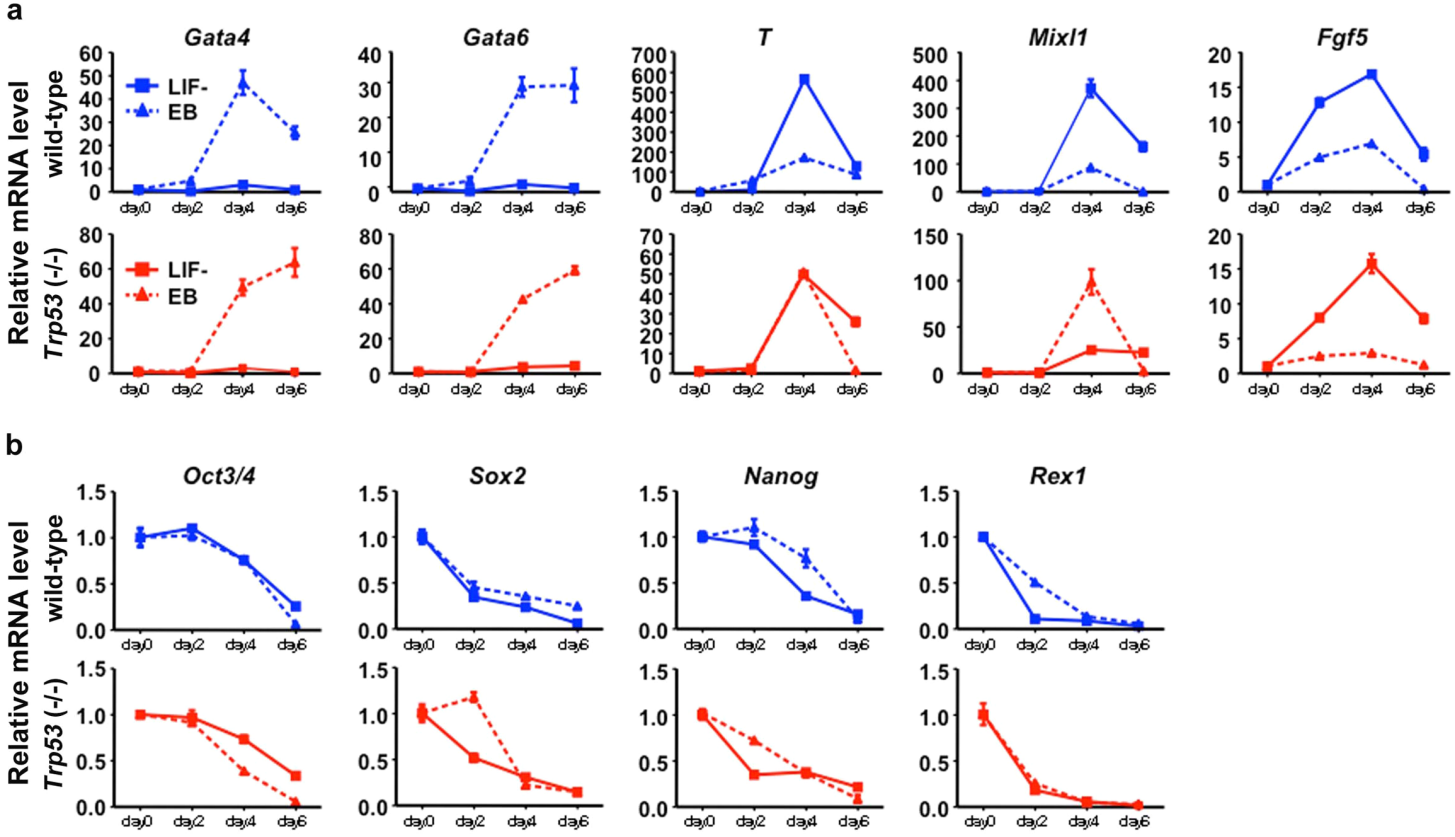
Differentiation of *Trp53*-null ES cells in three-dimensional culture. q-PCR analyses of gene expression profiles during differentiation of wild-type (blue) and *Trp53*-null (red) ES cells during the formation of EB in three-dimensional culture without LIF (dotted line). Transcript levels of the differentiation marker genes (a) and the stem cell marker genes (b) were evaluated at 2, 4, 6 day after withdrawal of LIF and normalized to the amount of *Gapdh* mRNA. Results are plotted as the expression level relative to that of day 0 (set as 1.0) with the mean ± SD of one biological sample assayed in three experiments.

The differentiation event induced by the withdrawal of LIF on two-dimensional dish surface is somewhat artificial. In contrast, the differentiation of mouse ES cells in floating culture to form the embryoid bodies (EB) is regarded as mimicking the event in normal development^32^, encouraging us to test the ability of differentiation of the *Trp53*-null ES cells in this context. The *Trp53*-null ES cells were cultured in a hanging drop to form EB and the regulation of the marker gene expression was examined. Surprisingly, the up-regulation of *T* and *Mixl1* occurred in the similar kinetics in EB derived from the *Trp53*-null ES cells to that of the wild-type ES cells (Fig. 5a). Both *Gata4* and *Gata6* expressions were also considerably recovered in the *Trp53*-null EB differentiation to the same level as in the EB derived from wild-type cells. The kinetics of the down-regulation of *Nanog* was also indistinguishable between the EB derived from the *Trp53-*null and wild-type ES cells (Fig. 5b). Therefore, the *Trp53*-null ES cells retained the normal ability to form EB as the wild-type ES cells and its defect in differentiation was context-dependent.

## Discussion

The process of mouse development is largely divided into two stages by the event to attach maternal uteri, implantation. At the pre-implantation development, the zygote develops into the blastocyst by generating two extraembryonic cell lineages, trophectoderm and extra-embryonic endoderm, which contribute to implantation. After implantation, the epiblast that consists of pluripotent cells form primitive ectoderm then undergo embryonic development^33^. Mouse ES cells derived from the ICM of blastocysts mimic the character of epiblast cells at early post-implantation embryos^34^. Since they undergo differentiation into the three germ layers and germ cells *in vitro*, they are regarded as a good model system of peri-implantation development that is hard to be analyzed *in vivo* due to the small size embryos with inaccessibility^35^,^36^. Here we demonstrated that *Trp53*-null ES cells showed defects in two-dimensional differentiation *in vitro* but differentiate normally in three-dimensional differentiation *in vitro* as well as in chimeric embryos after injection into blastocysts, indicating the difference of these contexts on the requirement of autonomous gene function.

Differentiation of ES cells on flat surface has several advantages to analyze the cell-autonomous gene function and the effect of extrinsic factors on particular differentiation process as well as to obtain synchronous differentiation into particular cell types^37^. When ES cells were cultured on gelatin-coated surface without LIF, the primitive ectoderm marker *Fgf5*^31^ was up-regulated at day 1 followed by the up-regulation of the mesoderm marker *T* at day 2, suggesting the sequential differentiation of ES cells as found in embryonic development, which mimic the sequence of differentiation events *in vivo*. It was reported that ES cells undergo selective differentiation to neuroectoderm in serum-free culture^38^ and lateral plate mesoderm on the surface coated by type IV collagen^37^, suggesting the similarity between *in vitro* differentiation and the developmental process *in vivo* can be extended to the later developmental stages. However, in our observation the requirement of *Trp53* for the differentiation event in a two-dimensional culture was clearly distinct from that in a three-dimensional culture *in vitro* or in a chimera formation *in vivo*. In the absence of *Trp53*, the induction of *T* was impaired in a two-dimensional culture but not in a three-dimensional culture; it should also be normal *in vivo* since *Trp53*-null ES cells contributed to the mesoderm lineage in chimeric embryos. Therefore, the differentiation of ES cells in a two-dimensional culture is somewhat artificial although it remains unclear what the differences from embryonic development are. Indeed, differentiation of cardiomyocytes is easily induced by three-dimensional culture but not in two-dimensional condition^39^, suggesting their difference in mesoderm differentiation. Adequate attention should be paid for the interpretion of the data obtained using these systems.

Here we showed an unequivocal result about the function of *Trp53* in mouse ES cells saying that *Trp53* is dispensable for the maintenance of pluripotency and the induction of differentiation. Moreover, the role of Trp53 for the repression of *Nanog* should be a minor effect except the DNA damage response as found in Fig. 1d because *Nanog* was expressed heterogeneously in undifferentiated ES cells and down-regulated in differentiating ES cells without *Trp53*. It was recently reported that the auto-repression of *Nanog* is the major mechanism to generate the heterogeneity in undifferentiated ES cells^40^, suggesting the minor role of Trp53 on the repression of *Nanog*. However, it does not mean that *Trp53* has no function in ES cells. Indeed, many *Trp53*-null ES cell lines we established showed abnormal karyotype, indicating the role of *Trp53* as a major guardian of the genomic stability. A few *Trp53*-null ES cell lines kept normal karyotypes, suggesting that a functional compensation for the loss of *Trp53* occurred in these cells. A previous report indicated that *Trp73* partly share the function with *Trp53* since Trp73 was essential for suppressing polyploidy and aneuploidy when Trp53 was inactivated^41^. To reveal the overlapaped function of *Trp53* and *Trp73* in ES cells, it will be required to generate double knockout lines of both genes probably by an inducible-knockout strategy to avoid the genetic instability during serial gene manipulation.

## Methods

### Cell culture and differentiation

ES cell lines (EB5) were cultured without feeder cells in Glasgow minimal essential medium (GMEM; Sigma) supplemented with 10% fetal calf serum, 1 mM sodium pyruvate, 10^−4^ M 2-mercaptoethanol, 1× nonessential amino acids, and 1,000 U of leukemia inhibitory factor (LIF) per ml on gelatin-coated dishes. For induction of differentiation in two-dimensional culture, ES cells were seeded (1,000 cells/cm^2^) without LIF on gelatin-coated dishes. Embryoid bodies were generated by the culture of 300 ES cells in a 15 μl drop of the culture medium without LIF.

### Quantitative PCR analysis

To quantify the levels of mRNA transcripts, cDNA were synthesized from 1 μg of total RNA using ReverTra (Toyobo), and quantified by real-time PCR using a CFX384 system (BioRad). Utilized primers were listed on supplemental Figure 3. All samples were tested in triplicate, and the mean relative amounts of each transcript were calculated by normalization to an endogenous control *Gapdh*. Results are given as the mean with ±SD. Statistical analysis was conducted using Student t-test. P < 0.05 was considered significant.

### Immunohistochemistry and Western blot

Cells were fixed by 4% paraformaldehyde in phosphate-buffered saline (PBS) for 30 min at 4°C and then permeabilized by 0.1% Triton X-100 in PBS for 15 minutes at room temperature (RT). After brief washing with PBS followed by blocking with PBS containing 2% FCS, the cells were incubated with following primary antibodies: anti-Trp53 mouse monoclonal antibody (1C12, Cell Signaling Technology), mouse anti-Pml mouse monoclonal antibody (Upstate Biotechnology), anti-T goat polyclonal anitibody (Santa Cruz Bitotechnology), anti-Gata4 goat polyclonal anitibody (Santa Cruz Biotechnology), and anti-Nanog rat monoclonal antibody (R&D) for overnight at 4°C. After washing with PBS, The cells were incubated with Alexa Fluor 488- or 555- or 633-conjugated donkey antibodies (Invitrogen) were used in a proper combination of species specificity as indicated in Figure legends. The fluorescent images were captured with an IX51 microscope with DP70 digital camera (Olympus) or a Leica SP2 confocal microscope (Leica).

Western blot was performed with anti-Trp53 mouse monoclonal antibody (1C12, Cell Signaling Technology) for the total cell lysates of wild-type and *Trp53*-null ES cells.

### Gene targeting of *Trp53*

For generation of Trp53-KO vector, genomic DNA fragment for 5′ and 3′ homology arms (Chr:11, 69583689-69587696 and 69591432-69595365 in GRCm38) were amplified from the EB5 genomic DNA using the primer pairs 5′-aatgtcgaccggt (*Sal I*, *AgeI*) AACAGTCTTAAACCAGATGTGGTGGCTC-3′ and 5′-aatctagaagatct (*Xba I*, *Bgl II*) CAGAGAAAAAGAGGCATTGAAAGGTC for 5′ homology arms, and 5′-aaatctagaagat (*XbaI*, *Bgl II*) CTGCCTCTGCATCCCGTCCCCATCACC-3′ and 5′-aatgcggccg (*Not I*) CTTTCTCTTTTGGTGATAGTACTGGGTGG-3′ for 3′ homology arms, respectively. The PCR product of 5′ homology arm (4.0 kb) was digested by *Sal I* and *Xba I*. That of 3′ homology arm (3.9 kb) was digested by *Xba I* and *Not I*. These DNA fragments were sub-cloned into the *Sal I* and *Not I* site of pBR-Blue II (pBR322 –derived plasmid carrying multicloning sites from pBluescript), resulting in pBR Blue II 5′ + 3′. For drug selection, the *Frt* flanked *SA-IRES-βgeo* selection cassette^24^ was inserted into *Bgl II* sites of pBR Blue II 5′ + 3′, resulting in a promoter-trap Trp53 KO vector. The *100 μg of* plasmid DNA was linealized by *NotI* and introduced into 10^7^ ES cells in 0.4-cm cuvette by electroporation with a Gene Pulser (Bio-Rad, 800 V, 3 μF) followed by the culture with selection drug, G418 (160 μg/ml, Sigma) for 7 to 10 days. G418-resistent colonies were picked up and the first allele targeting was confirmed by genomic PCR. The inserted FRT cassette was removed by subsequent transfection with the *FLPe* expression vector. This strategy was repeated for the targeting of the second allele to create homozygous *Trp53* KO ES cells.

### Karyotyping

Trypsin/EDTA-dissociated ES cell pellet was resuspended in 0.075 M KCl hypotonic solution and incubated at RT for 10 min. After that, the equal volume of Carnoy's fixative (acetic acid:methanol = 1:3) was added and then centrifuged (1,000 rpm × 5 min). After removal of supernatant, Carnoy's fixative was added and then centrifuged (1000 rpm × 5 min). This was repeated once and resuspended in Carnoy's fixative. Cell suspensions were dropped onto 50% ethanol-washed slides and dried overnight at RT. After chromosome staining with Hoechst33342, the slides were mounted with coverslips. The karyotype was evaluated by counting unscattered metaphase spreads with microscopic imaging with a 100x/NA1.4 oil immersion lens (UPLSAPO, Olympus).

### Production of chimeric embryos

For assessing *in vivo* contribution, *Trp53* (+/−) and *Trp53* (−/−) ES cells were transfected with pPB-CAG-Egfp-IZ using the piggy-Bac transposon system^42^ and the clones expressing Egfp homogeneously were selected. To obtain chimeric embryos, these cells were injected into C57/BL6J blastocysts. Embryos were dissected at 14.5 dpc and Egfp signals were detected under fluorescent dissecting microscope. All animal experiments were performed according to the guidelines for animal experiments of RIKEN Center for Developmental Biology and approved by the Animal Experiment Committee of the RIKEN Kobe Institute.

## Author Contributions

H.N. designed the experiments. M.S., S.O., S.F., K.M., S.T., M.Y. and H.N. performed the experiments. M.S. and H.N. wrote the manuscript with discussion of all other authors.

## Supplementary Material

Supplementary Information Supplementaly data
