# Supplementary material for: Maintenance of pluripotency in mouse ES cells without Trp53
Source: Sci Rep. 2013 Oct 15;3:2944. doi: 10.1038/srep02944 (PMC3796736; doi:10.1038/srep02944)
Supplement: Supplementary Information — Supplementaly data [file srep02944-s1.pdf]

# Maintenance of pluripotency in mouse ES cells without *Trp53*

Masaki Shigeta, Satoshi Ohtsuka, Mariko Yamane, Satomi Torikai-Nishikawa, Setsuko Fujii, Kazuhiro Murakami and Hitoshi Niwa

Information for supplemental data

**Supplemental Figure 1** (related to Fig. 3) Conversion of cell-cycle pattern in wild-type and *Trp53*-null ES cells. The tandem Fucci expression vector consists of the fusion of Gem-AG and Cdt1-mKO2 with P2A autocleavage site under the control of the CAG promoter was introduced into *Trp53 (+/-)* and *Trp53 (-/-)* ES cells by Piggy-bac transposon system and three independent clones were analyzed for each genotype. (a) Photomicrographs of the ES cells cultured without LIF for 5 days. (b) FACS analysis of the representative clones from each genotype cultured with or without LIF for 5 days. Comparable increase of G1 fraction was observed for both genotypes.

**Supplemental Figure 2** (related to Fig. 2) Western blot analysis of Trp53 expression in wild-type and *Trp53 (-/-)* ES cells. Lanes 1 and 2 were shown in Fig. 2c.

**Supplemental Figure 3** (related to Fig. 3 and 5) List of q-PCR primer sets.

**Supplemental Figure 4** (related to Fig. 4) Efficiency of chimera production with *Trp53*-null ES cells. ES cell clones were used to generate chimeric embryos by blastocyst injection. Injected blastocysts were transferred into the uterus of pseudopregnant ICR female. At 14.5 dpc, mice were sacrificed to collect embryos toevaluate chimerism by the expression of Egfp. The embryos obtained by injection of clone 22-5-3 (+/-) and 35-5-1 (-/-) were represented in Fig. 4.

# Supplemental Figure 1

*Shigeta et al*

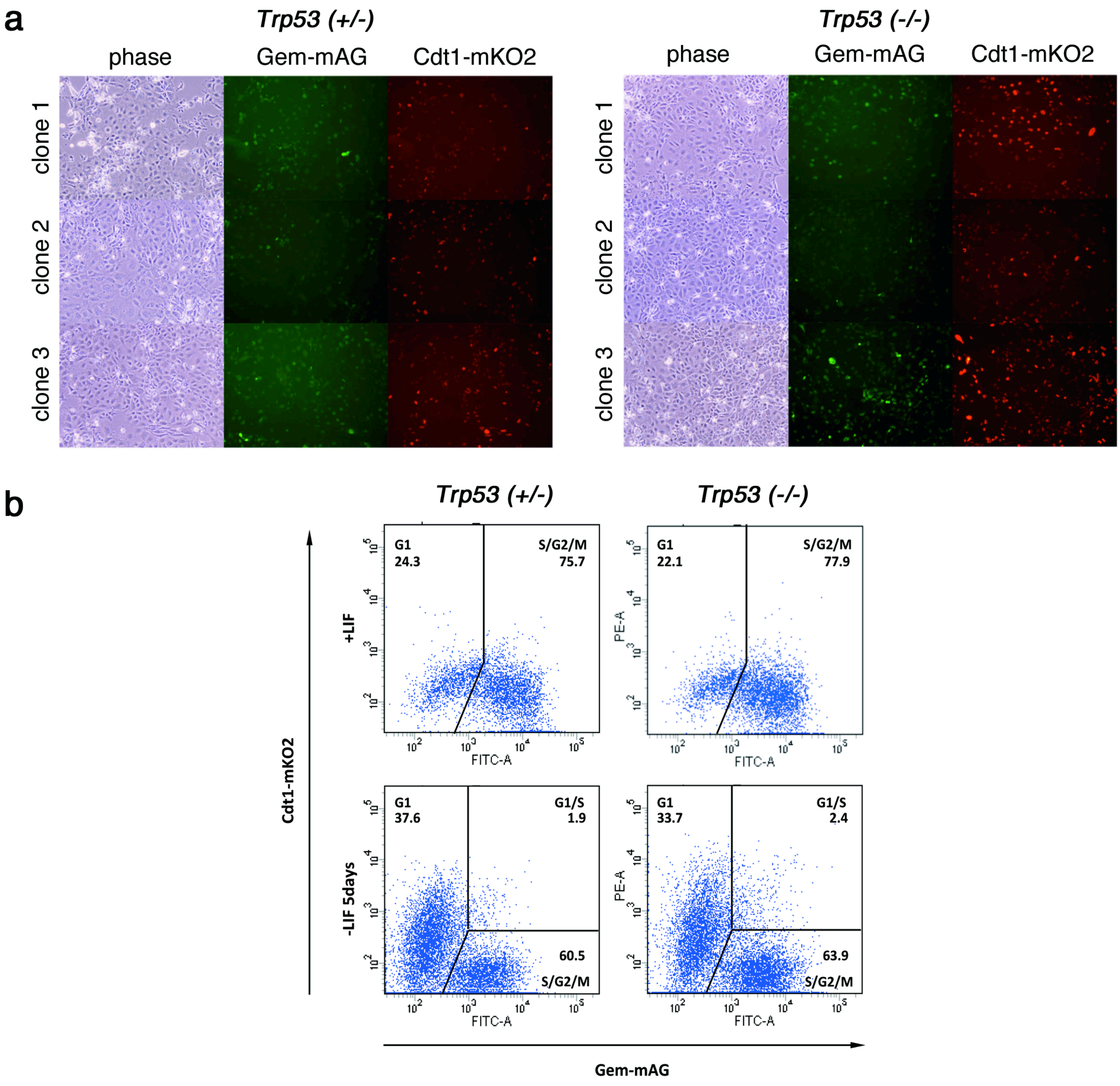

Supplemental Figure 2 *Shigeta et al*

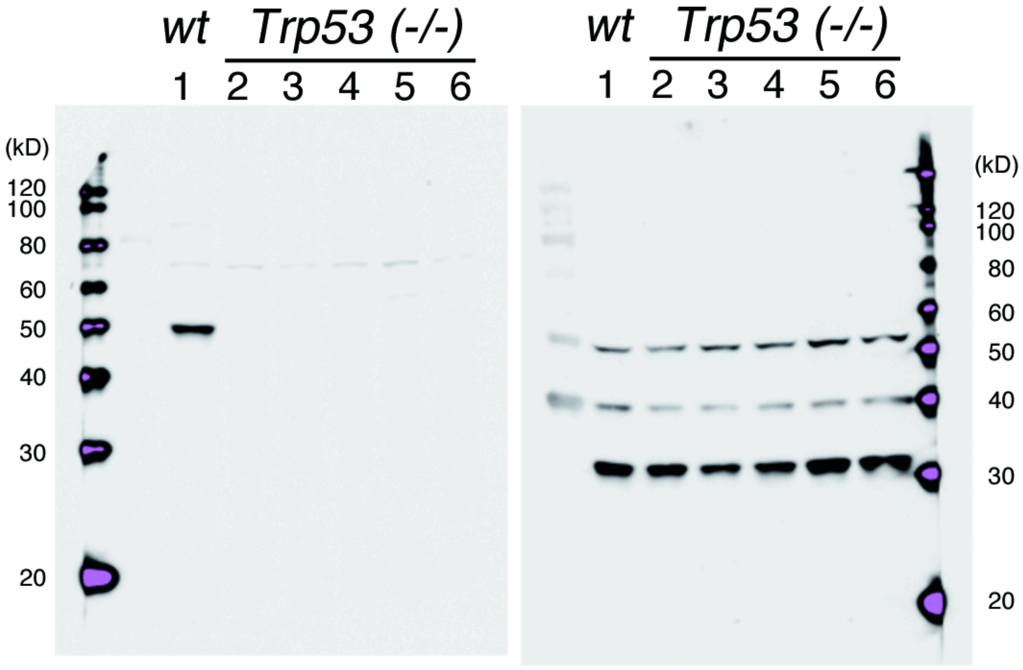


blot: α-Trp53 blot: α-Cdk2

| Target gene | Forward primer | Reverse primer |
|---|---|---|
| *Oct3/4* | 5’-cacgagtggaaagcaactca-3’ | 5’-agatggtggtctggctgaac-3’ |
| *Sox2* | 5’-gagtggaaacttttgtccgaga-3’ | 5’-gaagcgtgtacttatccttcttcat-3’ |
| *Nanog* | 5’-ccaggttccttccttcttcc-3’ | 5’-ggtgagatggctcagtggat-3’ |
| *Rex1* | 5’-gacagaggcactggggatac-3’ | 5’-cgtatgcaaaagtccccatc-3’ |
| *T* | 5’-aactttcctccatgtgctgagac-3’ | 5’-tgacttcccaacacaaaaagct-3’ |
| *Mixl1* | 5’-actttccagctctttcaagagcc-3’ | 5’-attgtgtactccccaactttccc-3’ |
| *Gsc* | 5’-gaagccctggagaacctctt-3’ | 5’-ccgagtccaaatcgcttta-3’ |
| *Lhx1* | 5’-aaggagcgaaggatgaaaca-3’ | 5’-cttgcgggaagaagtcgtag-3’ |
| *Gata4* | 5’-cccttccctcttcaaattcc-3’ | 5’-cttttccagagctccacctg-3’ |
| *Gata6* | 5’-gagctggtgctaccaagagg-3’ | 5’-tgcaaaagcccatctcttct-3’ |
| *Fgf5* | 5’-attagtggctgggctcaatg-3’ | 5’-agggggcagataaaaggaga-3’ |
| *Trp53* | 5’gcgtaaacgcttcgagatgt-3’ | 5’-ccccactttcttgaccattg-3’ |
| *Gapdh* | 5’-accacagtccatgccatcac-3’ | 5’-tccaccaccctgttgctgta-3’ |

| *Trp53* | Cell line | Number of EGFP(+) embryos | Number of collected embryos | Number of injected blastocysts |
|---|---|---|---|---|
| +/- | 22-5-2 | 3 | 15 | 132 |
| +/- | 22-5-3 | 17 | 31 | 68 |
| +/- | 22-5-4 | 4 | 22 | 64 |
| -/- | 35-5-1 | 1 | 2 | 40 |
| -/- | 35-5-3 | 1 | 2 | 40 |
| -/- | 35-5-4 | 0 | 1 | 40 |
